# Perinatal Arterial Stroke Treated With Stromal Cells Intranasally: 2-Year Safety and Neurodevelopment

**DOI:** 10.1161/STROKEAHA.125.050786

**Published:** 2025-07-14

**Authors:** Nienke Wagenaar, Lisanne M. Baak, Niek E. van der Aa, Floris Groenendaal, Jeroen Dudink, Maria Luisa Tataranno, Corine Koopman, Cornelia H. Verhage, Rian M.J.C. Eijsermans, Heleen C. van Teeseling, Liesbeth S. Smit, Reint K. Jellema, Timo R. de Haan, Hendrik J. ter Horst, Willem P. de Boode, Sylke J. Steggerda, Susanne M. Mulder-de Tollenaer, Koen P. Dijkman, Colin G. de Haar, Linda S. de Vries, Frank van Bel, Cobi J. Heijnen, Cora H. Nijboer, Manon J.N.L. Benders

**Affiliations:** Department of Neonatology (N.W., L.M.B., N.E.v.d.A., F.G., J.D., M.L.T., C.K., L.S.d.V., F.v.B., M.J.N.L.B.), University Medical Center Utrecht Brain Center and Wilhelmina Children’s Hospital, Utrecht University, the Netherlands.; Department for Developmental Origins of Disease (C.H.N.), University Medical Center Utrecht Brain Center and Wilhelmina Children’s Hospital, Utrecht University, the Netherlands.; Child Development and Exercise Center (C.H.V., R.M.J.C.E.), University Medical Center Utrecht, Utrecht University, the Netherlands.; Department of Pediatric Neuropsychology (H.C.v.T.), University Medical Center Utrecht, Utrecht University, the Netherlands.; Department of Pediatric Neurology, Erasmus MC-Sophia Children’s Hospital, Rotterdam, the Netherlands (L.S.S.).; Department of Neonatology, Maastricht University Medical Center, the Netherlands (R.K.J.).; Department of Neonatology, Emma Children’s Hospital, Academic Medical Center, Amsterdam University Medical Center, the Netherlands (T.R.d.H.).; Department of Neonatology, Beatrix Children’s Hospital, University Medical Center Groningen, University of Groningen, the Netherlands (H.J.t.H.).; Department of Neonatology, Radboud University Medical Center, Radboud Institute for Health Sciences, Amalia Children’s Hospital, Nijmegen, the Netherlands (W.P.d.B.).; Department of Neonatology, Willem-Alexander Children’s Hospital, Leiden University Medical Center, the Netherlands (S.J.S.).; Department of Neonatology, Isala Clinics, Zwolle, the Netherlands (S.M.M.-d.T.).; Department of Neonatology, Maxima Medical Center Veldhoven, the Netherlands (K.P.D.).; Cell Therapy Facility, Pharmacy Department, University Medical Center Utrecht, the Netherlands (C.G.d.H.).; Department of Psychological Sciences, Rice University, Houston, TX (C.J.H.).

**Keywords:** cerebral palsy, child development, infant, newborn, ischemic stroke, mesenchymal stem cells

## Abstract

**BACKGROUND:**

The PASSIoN study (Perinatal Arterial Stroke Treated With Stromal Cells Intranasally) demonstrated the feasibility and short-term safety of single-dose allogeneic mesenchymal stromal cells (MSCs) administered intranasally to neonates with perinatal arterial ischemic stroke between February 2020 and April 2021. In this study, we assessed long-term safety and neurodevelopmental outcomes and explored outcome differences with a non–MSC-treated cohort.

**METHODS:**

In this post hoc analysis, we evaluated the safety of MSC administration by assessing brain tissue loss, adverse events, and neurodevelopmental outcomes of PASSIoN participants (N=10). The tissue loss ratio was calculated using semi-automatic segmentation based on neonatal and 3-month magnetic resonance imaging. At the age of 2 years, we assessed the occurrence of cerebral palsy, motor and cognitive delays (*Z* score <−1 SD), behavioral and language problems, visual field defects, and epilepsy. We selected a non–MSC-treated registry cohort (N=39) born between 1994 and 2022, who would have met PASSIoN trial inclusion criteria to compare magnetic resonance imaging and outcome characteristics.

**RESULTS:**

At 3 months, the mean±SD tissue loss ratio of PASSIoN participants was 89±21%, indicating more preserved tissue than expected based on initial stroke volume. By the age of 2 years, no related adverse events were reported. Two children (20%) developed cerebral palsy (Gross Motor Function Classification System I) without motor developmental delays. Cognitive, behavioral, and language problems affected 10% to 20%, and none had developed epilepsy. Compared with the registry cohort, and PASSIoN participants showed less often asymmetry of the posterior limb of the internal capsule (40% versus 81%; *P*=0.02) and the cerebral peduncle (10% versus 61%; *P*=0.01) on 3-month magnetic resonance imaging and had a better motor performance at the age of 2 years (median [interquartile range] *Z* score, 0.3 [0.8] versus −0.4 [1.5]; *P*=0.003).

**CONCLUSIONS:**

This study demonstrates the long-term safety of intranasal MSC therapy in 10 infants with perinatal arterial ischemic stroke and may suggest better motor outcomes compared with the literature and a non–MSC-treated cohort. Randomized controlled trials are required to confirm MSC efficacy for children with perinatal arterial ischemic stroke.

**REGISTRATION:**

URL: https://www.clinicaltrials.gov; Unique identifier: NCT03356821.

Perinatal arterial ischemic stroke (PAIS) occurs in 1 in 5000 to 10 000 newborns.^1^ Depending on the extent and location of the stroke, PAIS can lead to lifelong deficits in, for example, motor and cognitive functioning, significantly impacting the children’s quality of life.^2,3^ Developing new therapies for patients with PAIS is urgently needed, as no curative treatments are currently available.

**See related article, p 2419**

Emerging evidence highlights the neuroregenerative potential of mesenchymal stromal cell (MSC) therapy for perinatal brain injury. In animal models, intranasal MSC therapy has proven effective in reducing tissue loss and improving long-term functional outcomes.^4–7^ Most in-human trials describe MSC administration for treatment of established cerebral palsy (CP), which is often a result of perinatal brain injury. In an individual participant data meta-analysis, children aged 0 to 18 years with CP showed improved motor function 6 months after infusions of umbilical cord (UC) blood, a rich source of MSCs.^8^ In addition, structural improvements of the corticospinal tracts (CSTs) measured by diffusion tensor imaging were observed 12 months after intrathecal UC-MSC treatment in 4- to 14-year-old children with CP.^9^

While these in-human trials indicate that MSC therapy can be neuroregenerative years after injury, preclinical studies suggest that administering MSCs shortly after injury onset may be more beneficial.^7^ The neurogenic capacity of the brain is highest early in life,^10^ and in preclinical studies, MSCs migrate more effectively to the lesion site where high levels of chemotactic cues are present shortly after PAIS onset.^11,12^ Intranasal administration provides a noninvasive, clinically applicable route to target the neonatal brain.^6,7,13^ In the phase I PASSIoN trial (Perinatal Arterial Stroke Treated With Stromal Cells Intranasally; URL: https://www.clinicaltrials.gov; Unique identifier: NCT03356821), we demonstrated the feasibility and safety up to 3 months of age of administering intranasal MSCs within 1 week after birth in 10 infants with PAIS.^14^ Based on animal studies, we expect that in these infants, intranasal-MSC administration will dampen inflammation and stimulate neuroregeneration leading to decreased brain tissue loss and improved long-term neurodevelopmental outcomes.^6,7^ Although the trial was not designed to assess efficacy, the promising results showed that 70% of the PASSIoN infants had a low risk of developing CP based on a qualitative follow-up magnetic resonance imaging (MRI) and early motor assessments at 3 to 4 months of age, while all infants were initially considered at high-risk based on the extent and localization of injury.^14^

The goal of the current study is to assess the long-term safety of intranasal-MSC treatment by reporting the neurodevelopmental outcomes of the 10 study participants at 2 years of age. As an additional safety measure, we quantitatively analyzed brain tissue loss after PAIS using serial MRI. In an exploratory analysis, we compared MRI and neurodevelopmental outcomes of the PASSIoN participants with a non–MSC-treated registry cohort.

## Methods

### Study Design and Participants

In the PASSIoN trial, 10 neonates were prospectively enrolled in a nationwide phase I open-label intervention study after written parental consent between February 11, 2020, and April 29, 2021.^14^ The study was approved by the Dutch Central Committee on Research Involving Human Subjects (NL59265.000.16). Participants were born ≥36.0 weeks of gestation and had MRI-confirmed predominantly unilateral PAIS in the middle cerebral artery region including the cortex, white matter, CSTs, and basal ganglia adhering to the trial’s inclusion criteria. Neonates received 50×10^6^ bone marrow–derived MSCs intranasally within 7 days of presenting with signs suggestive of PAIS at the neonatal intensive care unit of the Wilhelmina Children’s Hospital, Utrecht, the Netherlands.^14^ We previously reported the trial’s primary outcomes’ feasibility and (sub)acute safety, together with qualitative analysis of the neonatal and a 3-month MRI scan, and neurodevelopment at 4 months.^14^ In this post hoc analysis, we report possible (serious) adverse events that occurred after 3 months of age, 2-year outcomes of these patients collected during clinical neurodevelopmental follow-up, and a quantitative tissue loss analysis using serial MRI as an additional safety measure. This study followed the Strengthening the Reporting of Observational Studies in Epidemiology reporting guidelines. Data collected for the study cannot be made available to others because the parents of participants did not give consent to share data with other parties.

As an exploratory analysis, we compared the MRI and neurodevelopmental outcomes with a cohort from the Neonatal Stroke Registry Utrecht, which contains clinical, neuroimaging, and outcome data of children admitted to the Wilhelmina Children’s Hospital with PAIS from 1990 onward. We selected a subset of infants (N=39) who would have been eligible to participate in the PASSIoN trial based on the inclusion and exclusion criteria if the trial had been enrolling participants at the time of their birth. A part of this registry cohort (n=12/39, 31%) received 3000-IU/kg erythropoietin intravenously in the neonatal period in light of their participation in a phase I safety and feasibility intervention trial.^15^ Parents and patients (depending on their age) of the PASSIoN and registry cohort gave written consent for data use in the Neonatal Stroke Registry Utrecht.

### MRI Characteristics

Infants in the PASSIoN and registry cohort underwent a neonatal cranial MRI, performed within 10 days of symptom onset, and a follow-up MRI around 3 months of age as part of standard care. Scans were performed on a 1.5T or 3T Tesla Philips whole-body Achieva system including an 8-channel head coil (Philips Medical Systems, Best, the Netherlands), including 2 mm axial T1-weighted imaging and T2-weighted imaging (T2WI), and 3-mm diffusion-weighted imaging (scanning characteristics were reported previously).^16^ During MRI, infants wore hearing protection, were wrapped in a vacuum pillow to avoid movement, and received the mild sedative choral hydrate orally (50 mg/kg) with vital parameters monitored.

### MRI Analysis

The affected arterial territory and the involvement of (motor-related) brain structures (posterior limb of the internal capsule and cerebral peduncle) were qualitatively scored as previously described.^14^ In addition, we qualitatively assessed stroke involvement in the perirolandic area, where the primary motor and sensory cortices are located. We scored the perirolandic area as injured when either clear diffusion restriction was visible on neonatal DWI or hyperintensity on neonatal T2WI, or tissue loss on the follow-up T2WI sequence.

The PASSIoN study protocol included predefined safety stopping rules; all but 1 were previously reported.^14^ The final rule called for stopping if the infarcted area increased by >20% after MSC administration in 3 patients, indicated by a tissue loss ratio (TLR) >120%. We assessed TLR in the PASSIoN cohort by comparing neonatal stroke and brain volumes with 3-month brain volumes (see Supplemental Material for calculation methods)^17^ but could not repeat this for the registry cohort due to variability in scan quality and protocols over time. Brain tissue and stroke volumes were calculated based on neonatal DWI and T2WI sequences using a convolutional neural network segmentation method.^18^ Segmentations were visually checked and manually adjusted with ITK-SNAP (version 3.8.0). Stroke volume was expressed relative to the total brain volume (excluding cerebrospinal fluid and ventricles).

### Neurodevelopmental Assessments

All patients with perinatal brain injury receive routine clinical follow-up visits at 3 to 4, 9 to 15, and 24 months of age at the neonatology outpatient clinics of the Dutch neonatal intensive care units. During the visit at the age of 2 years, the patient was assessed by a neonatologist, a pediatric physiotherapist, and a pediatric psychologist, who performed a neurodevelopmental examination including the Gross Motor Function Classification System classification in the case of CP and either the Bayley Scales for Infant and Toddler Development, version 3 (>2009) or the Griffiths Scales of Child Development third Edition (Neonatal Stroke Registry Utrecht participants born before 2009). Language delays were assessed with a standardized parent-proxy lexical development questionnaire measuring vocabulary comprehension and production or by assessment of the neonatal follow-up team. Behavioral problems were identified with the Child Behavior Checklist, and visual fields were examined >9 months by an ophthalmologist in case of MRI-diagnosed optic radiation injury. PASSIoN participants also underwent the Hammersmith Infant Neurological Examination. These assessments provided information on motor, cognitive, language, behavioral performance, and the presence of CP, epilepsy, and visual field defects. The occurrence of serious adverse events was monitored by asking parents about prescribed medication, hospital admissions, and treatments by other health care professionals. All continuous parameters were converted to *Z* scores to allow comparison of different assessment methods. A *Z* score <−1 was considered a developmental delay, similar to a previously published study from our group.^2^

### Statistical Analysis

We tested the data for normality by visual inspection, the Levene test for normality, and the Shapiro-Wilk test. Because of the small sample size, we handled missing data by pairwise deletion. Continuous parameters were depicted as mean and SD or median and interquartile ranges, and categorical parameters were depicted as numbers and percentages. We compared clinical and MRI characteristics of the infants from the PASSIoN cohort with the registry cohort by using either the independent samples *t* test, the Mann-Whitney *U* test, the Fisher Exact test, or the χ^2^ test, where applicable.

## Results

The baseline characteristics of the patients and short-term safety of MSC administration have been previously described.^14^

On neonatal MRI, the stroke volume ranged between 4 and 24% of total brain volume (median [interquartile range], 8% [8%]). As a safety measure, we assessed the TLR. A TLR over 100% indicates tissue loss beyond the initial stroke lesion and vice versa. The mean±SD TLR was 89±21%, and all children had a TLR below the predefined per-protocol stopping criterion of 120% (range, 55%–114%).

At 24.3 (0.9) months of age, 2 parents reported unplanned hospital visits with their child: 1 due to an episode of apnea caused by gastroesophageal reflux and 1 because of a febrile seizure during a viral infection. Both events were considered unrelated to their study participation. None of the children had used medication after the initial admission. Five (50%) children did not experience an adverse outcome on any of the assessed domains (Table 1). Two of 10 children (20%) developed CP. Both had a Gross Motor Function Classification System I classification. One child had mild bilateral CP affecting the right leg and the left arm due to bilaterally affected motor cortices on neonatal MRI; the other child had unilateral CP with full functionality but reduced movement quality of the hand. Four additional children (40%) exhibited mild muscle tone asymmetry, with a higher tone in 1 extremity contralesional to the affected hemisphere. While full functionality of all extremities was maintained, they showed a preference for using the ipsilesional hand. All children had a Bayley motor composite score >85 indicating normal motor development, and children started walking independently at a median age of 14 (3) months. Bayley cognitive composite scores were within the normal range for all but 1 child, who had a score of 82. Two children exhibited a language delay, and 1 child experienced behavioral problems consisting of sleeping problems. None were diagnosed with epilepsy or a visual field defect.

**Table 1.**
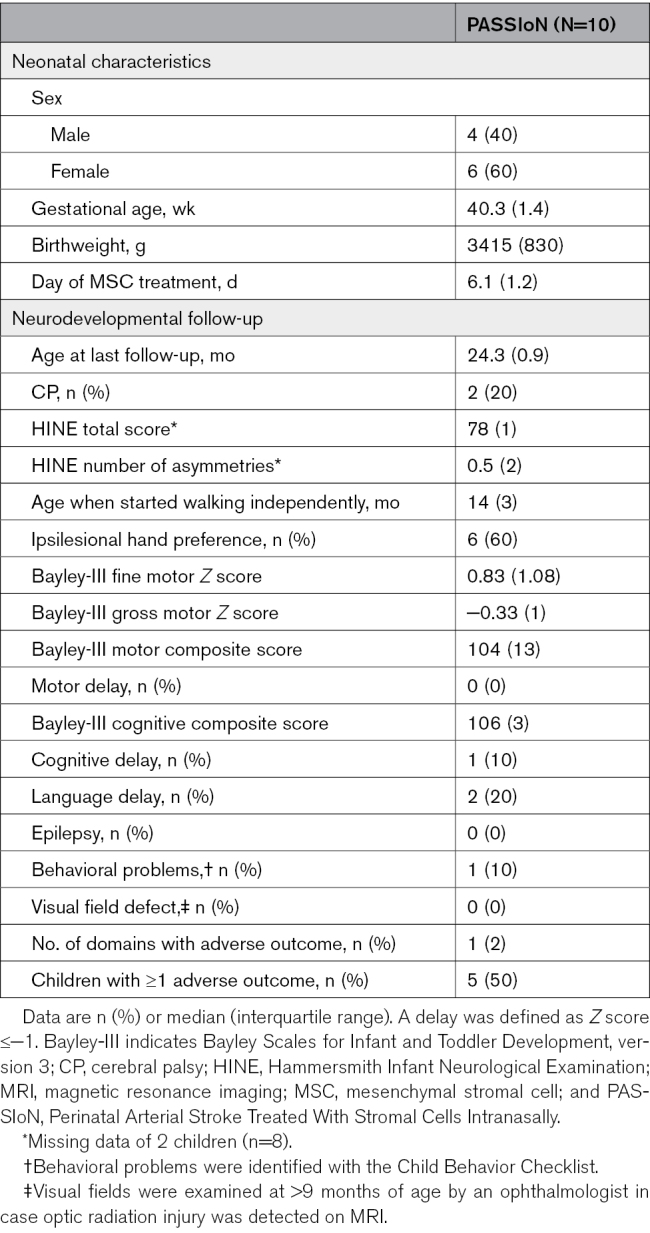
Baseline and Neurodevelopmental Outcome Characteristics of the 10 Infants Who Received Mesenchymal Stromal Cells Intranasally

### Comparison With Registry Cohort

As an exploratory analysis, MRI characteristics and neurodevelopmental outcome of the PASSIoN cohort were compared with a subset of 39 patients from our registry, born between 1994 and 2022 (Tables 2 and 3). We did not observe statistical differences between the neonatal characteristics of both cohorts except for birth year (Table S1). Of the registry cohort, 2 of 6 infants with perinatal asphyxia (PA) received therapeutic hypothermia (TH; the other 4 children were born before TH was available), while 1 infant of the PASSIoN cohort had mild PA without the need for TH.

**Table 2.**
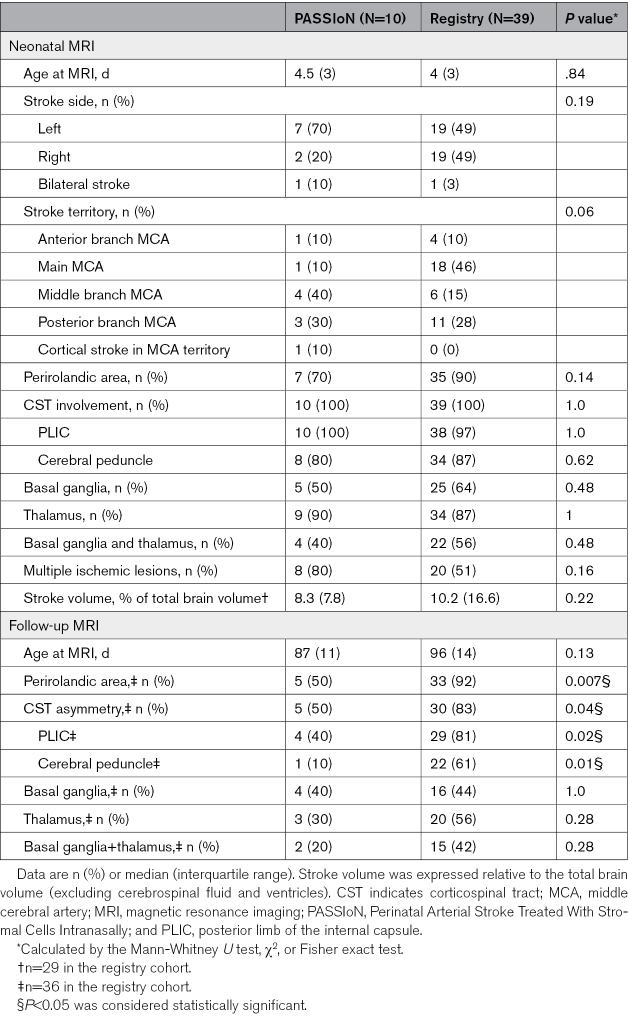
MRI Characteristics of the Infants Participating in the PASSIoN Trial and a Registry Cohort Adhering to the PASSIoN Trial Inclusion and Exclusion Criteria

**Table 3.**
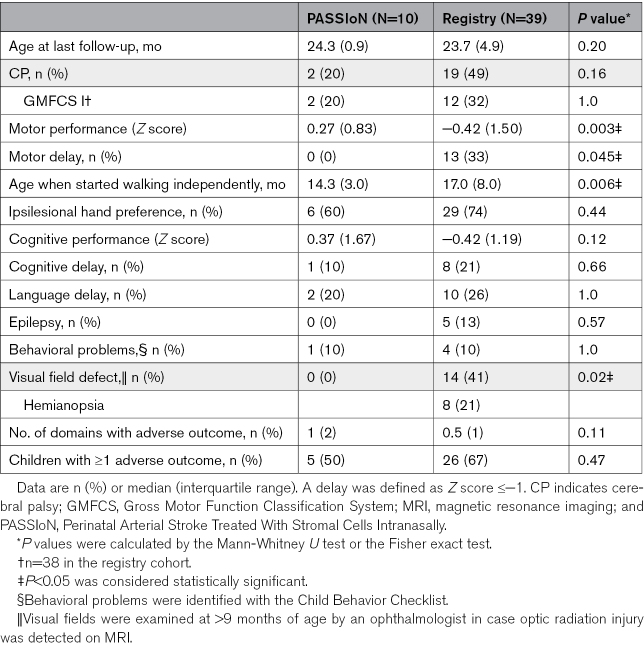
Neurodevelopmental Outcomes at 2 Years of Age of the Infants of the PASSIoN Cohort and a Registry Cohort Adhering to the PASSIoN Trial Inclusion and Exclusion Criteria

The cohorts underwent a diagnostic MRI at a median (interquartile range) of 4.5 (3) and 4 (3) postnatal days (PASSIoN and registry cohort, respectively; Table 2). In the PASSIoN cohort, patients more frequently had a middle branch middle cerebral artery stroke (40%), whereas, in the registry cohort, a main middle cerebral artery stroke was most common (46%). Differences in stroke territory distribution and stroke volume between the 2 cohorts did not reach statistical significance (*P*=0.06 and 0.22, respectively). On the neonatal MRI, no statistical differences were observed concerning the involvement of the perirolandic cortex or the CST, areas highly predictive of motor development. At the follow-up MRI (postmenstrual age of PASSIoN and registry cohort: 53 (2) and 53 (3) weeks), patients in the PASSIoN cohort less frequently showed tissue loss in the perirolandic region (50% versus 92%; *P*=0.007) and asymmetry in the posterior limb of the internal capsule (posterior limb of the internal capsule; 40% versus 81%; *P*=0.02) and the cerebral peduncle (10% versus 61%; *P*=0.01) compared with the registry cohort, while no differences were observed in basal ganglia and thalamus asymmetry. Non-PAIS injuries (eg, additional ischemic lesions or global atrophy) were not significantly different between PASSIoN and the registry cohort on both MRI scans.

At neurodevelopmental follow-up at the age of 2 years, the rate of CP was 20% versus 49% (*P*=0.16) between the PASSIoN and registry cohort (Table 3). The motor performance score of the PASSIoN cohort was significantly higher than the registry cohort (median *Z* score 0.3 (0.8) versus −0.4 (1.5); *P*=0.003), with 0% and 33% of children having a motor delay, respectively. In addition, the scores of both cohorts were significantly different from 0 in a 1-sample *t* test (*P*=0.034 and 0.003, respectively). Children from the PASSIoN cohort started walking independently at a significantly younger age (*P*=0.006). Hand preference was similar, with 60 to 74% in both cohorts favoring the ipsilesional hand. Fewer children in the PASSIoN cohort had cognitive delays, and none developed epilepsy although rates were not statistically different. Visual field defects were more common in the registry cohort (0% versus 41%; *P*=0.02). The number of patients with a language delay or a behavioral problem did not differ. Because moderate to severe PA might affect neurodevelopment, we repeated the analyses excluding the 6 infants with PA from the registry cohort and found similar results (data not shown).

## Discussion

In this study, we showed that intranasal-MSC therapy after PAIS is safe up to 2 years of age. We did not observe any long-term negative effects, including serious adverse events in the 10 participants of the PASSIoN trial. The ratio of lost tissue after the stroke was 89% on a group level, which might indicate that more brain tissue was preserved at 3 months of age than would have been expected based on the initial stroke lesion, which may be considered an additional confirmation of safety. Furthermore, only 2 patients (20%) in the PASSIoN cohort developed CP (Gross Motor Function Classification System I) at the age of 2 years, and all MSC-treated patients had motor developmental scores within the normal range including the children with CP. Children also performed well regarding cognition, language, and behavior and did not show signs of epilepsy or visual field defects. In comparison to a registry cohort, PASSIoN participants showed less asymmetry of the CSTs on 3-month MRI and had better motor performance at the age of 2 years.

In preclinical studies, MSC therapy is a promising neuroregenerative treatment for perinatal brain injury. Due to the unmet need for effective therapies for children with perinatal brain injury, the safety and feasibility of MSC therapy in neonates have been investigated in several studies over the past decade. To date, 4 studies have demonstrated the safety and feasibility of intravenous UC cell and MSC administration to infants with hypoxic-ischemic encephalopathy due to PA treated with TH.^19–22^ In adults with stroke and children with CP (median age, 43 months), MSC and UC therapy via various invasive administration routes have been shown to improve motor function.^8,23^ The research field of MSC therapy for perinatal hypoxic-ischemic brain injury must now prioritize randomized controlled studies that evaluate efficacy.

### Comparison With Registry Cohort

As an exploratory analysis regarding the potential effect of intranasal-MSC therapy, we compared outcomes of the PASSIoN participants to a registry cohort that met the trial’s inclusion and exclusion criteria. We acknowledge that this comparison is not ideal to assess the potential efficacy of MSC therapy, as the trial was not designed accordingly. Nonetheless, we aimed to provide information that could be useful to plan next-phase trials.

Although initial CST involvement was similar between both cohorts, PASSIoN participants less often showed an asymmetry of the CSTs on MRI at 3 months and had better motor performance at the age of 2 years. The known CP rate observed in children with these types of stroke is 40% to 66% and aligns with the rate observed in our registry cohort.^24–26^ However, in PASSIoN infants, the rate of asymmetry of the CSTs and CP was remarkably lower: only 20% (n=2) developed CP, and 4 additional children demonstrated a mild tone asymmetry not classified as CP. We hypothesize that the MSC therapy might have stimulated neuroregeneration in the affected motor regions, thereby positively affecting motor outcomes and preventing CP. Although not statistically different from the registry cohort and all limitations of our current comparison considered, reducing CP from 40% to 66% to 20% could have an important clinical impact on children with PAIS and their families. Future MSC efficacy trials for perinatal brain injury could be powered using these rates.

The prevalence of cognitive deficits and epilepsy appeared lower in the PASSIoN cohort, though not significantly. In both cohorts, nonmotor deficits were less common and similar to the incidence reported in the literature.^2^ Longer follow-up may be needed to assess the impact of neuroregenerative therapies on these domains as nonmotor delays may emerge later in life. However, Giraud et al^27^ found that the age at which children with PAIS began walking correlated with cognition scores at the age of 7 years. Of note, visual field defects were significantly less present in the PASSIoN cohort. We hypothesize that this difference is likely due to stroke location: more patients in the registry cohort had PAIS involving the optic radiation region, and injury to this structure has been strongly linked to visual field defects.^28^

With few trials studying MSC therapy in neonates with brain injury, data on neurodevelopmental outcomes remain sparse. In a phase I trial, 6 infants with moderate to severe PA showed no signs of hypoxic-ischemic injury on MRI after receiving intravenous UC-derived MSCs during TH. At 12 to 17 months, all had average cognitive scores, and 5 had average motor scores.^21^ These findings align with ours and with preclinical studies, which showed a 40% to 50% improvement in motor and cognitive outcomes with intranasal-MSC therapy.^6,7^

### Factors Influencing MSC Efficacy

As described in animal studies, several factors might influence MSC therapy effectiveness including the type of brain injury and MSC source (eg, bone marrow and UC), timing (eg, shortly after birth or later in life), and route (eg, intravenous, intranasal, and intrathecal) of administration. Chemotactic and inflammatory mediators produced in reaction to PAIS or hypoxic-ischemic encephalopathy attract MSCs to damaged brain regions.^11,13^ Upon arrival, MSCs support neuroregeneration by secreting, for example, growth factors to boost endogenous neurogenesis, and immunomodulatory factors to dampen neuroinflammation. With this in mind, we speculate that MSC efficacy depends on the timing of administration, lesion severity (affecting signaling factor release), and the brain’s age-dependent neuroreparative capacity.^10^ Because MSCs are known to support repair by stimulating neural stem cells in the neurogenic niches,^13^ injury to these niches might reduce MSC efficacy. Therefore, neonates with a certain stroke subtype or location may benefit more than others. In adult stroke, lesion quantification is possible with, for example, computed tomography perfusion imaging.^29^ With the aim to quantify tissue loss after PAIS, we calculated the TLR for the PASSIoN cohort. We observed a wide variation in TLR; however, the small sample size and absence of reference values of untreated cohorts limit conclusions about differences between PAIS subtypes or locations.

### Limitations

This study has several limitations. First, the PASSIoN study had only 10 participants and was not designed to assess intranasal-MSC efficacy, limiting in-depth statistical analyses. Second, establishing a representative control cohort was challenging. We were not able to apply a matched case-control design due to significant variability in maternal, perinatal, and stroke characteristics, combined with the rarity of the condition. Children in the registry cohort had more main branch middle cerebral artery strokes; although the stroke volumes did not differ significantly between both cohorts, differences in location and extent of the stroke could have influenced functioning at 2 years of age. Furthermore, the registry cohort included several participants born before 2010 (Table S1), potentially introducing differences in clinical care, (early) rehabilitation strategies, and outcome measures such as the usage of the Griffiths and Bayley scales for developmental assessments. A subset participated in a phase I intervention trial where they received 3 doses of erythropoietin during the neonatal period.^15^ However, we speculate that the inclusion of erythropoietin-treated children in the registry cohort is more likely to underestimate the effect of intranasal-MSC therapy. We also found no evidence that the presence of neonates with PA in the registry cohort affected the outcome. Due to the small sample size, we could not statistically adjust for these differences, which might limit the generalizability of our findings and highlight the need for high-quality randomized controlled trials to study intranasal-MSC efficacy.

The TLR analysis has several limitations. Variations in scanning protocols and scan quality over time hindered reliable analyses of the registry cohort. Edema caused by the stroke lesion and scan timing may have affected stroke volume measurements as diffusion restriction could vary over time, especially in the penumbra of the lesion.^30^ For the calculations, we assumed that the volume of the contralesional hemisphere was unaffected by PAIS in the first 3 months of life, which is supported by our unpublished cohort data. Finally, larger strokes might create more space for remaining brain tissue to waiver, potentially overestimating brain volumes at 3 months. Despite these limitations, this represents the best available unique data given the rarity of the condition. Therefore, randomized controlled trials are essential to overcome these challenges.

### Conclusions

This study showed that intranasal-MSC therapy for infants with PAIS is safe up to 2 years of age. An increasing number of studies have demonstrated the safety of MSC therapy for perinatal brain injury via various administration routes. In our minimally invasive safety-feasibility study, explorative analyses showed promising results regarding the motor outcome of intranasal-MSC–treated patients, which was better compared with a registry cohort and to rates reported in the literature. Because no therapies are currently available, developing neuroregenerative therapies for infants with PAIS is crucial to improve the quality of life for both the children and their families. Future research should prioritize conducting randomized controlled trials to establish the efficacy of intranasal-MSC therapy for perinatal brain injury.

## Article Information

### Acknowledgments

Drs Wagenaar and Baak performed investigation, project administration, and formal analysis and wrote the original draft. Drs van der Aa, Groenendaal, Dudink, Tataranno, Koopman, Smit, Jellema, de Haan, ter Horst, de Boode, Steggerda, Mulder-de Tollenaer, and Dijkman, C.H. Verhage, R.M.J.C. Eijsermans, and H.C. van Teeseling aided in investigation. Dr de Haar conceptualized and performed preclinical investigations and provided resources by means of preparation of the mesenchymal stromal cell product and validation of the production process. Drs de Vries, van Bel, Heijnen, Nijboer, and Benders performed funding acquisition, conceptualization, and supervision. All authors reviewed and edited the article and confirm that they had full access to all the data in the study and accept responsibility to submit for publication.

### Sources of Funding

This study was supported by the Netherlands Organization for Health Research and Development, the Netherlands (Translational Adult Stem Cell [TAS] research grant 11600200). The funder of the study had no role in study design, data collection, data analysis, data interpretation, or writing of the report.

### Disclosures

Dr de Boode is the president of the European Society for Paediatric Research. Dr Groenendaal is an expert witness in medicolegal cases. The other authors report no conflicts.

### Supplemental Material

Supplemental Methods

Table S1

Figure S1
